# Patients’ quality of life improves after surgical intervention of stage III medication-related osteonecrosis of the jaw

**DOI:** 10.1007/s10006-020-00927-7

**Published:** 2020-11-23

**Authors:** Stefan Moll, Steffen Mueller, Johannes K. Meier, Torsten E. Reichert, Tobias Ettl, Christoph Klingelhöffer

**Affiliations:** Department of Cranio- and Maxillofacial Surgery, Hospital of the University of Regensburg, Franz-Josef-Strauß-Allee 11, 93053 Regensburg, Germany

**Keywords:** Bisphosphonates, Denosumab, Osteonecrosis, Surgery, Recurrence, Quality of life

## Abstract

**Purpose:**

The treatment of advanced stages of medication-related osteonecrosis of the jaw (MRONJ) remains challenging. In order to improve decision making concerning the therapy, we examined the change of patients’ quality of life (QoL) after surgical treatment of MRONJ stage III.

**Method:**

The primary outcome variable was patients’ QoL. It was preoperative (T0), 6 weeks postoperative (T1) and 6 months postoperative (T2) assessed by the European Organisation for Research and Treatment of Cancer QoL-H&N35 (EORTC QoL-H&N35) and the Oral Health Impact Factor-G14 (OHIP-G14) questionnaire in a prospective cohort study. Other variables included location, age, sex, risk factors, and recurrence. Descriptive statistics and general multivariate regression models were calculated.

**Results:**

Forty-three patients with stage III MRONJ underwent surgery. OHIP-G14 scores decreased (improvement) statistically significant (*p* = .001) by 52.02% (T0-T1) and 56.45% (T1–T2). EORTC QoL-H&N35 showed statistical improvement for “swallowing” (*p* = .007), “opening mouth” (*p* = .045), “painkiller” (.005), “weight loss” (.004), “pain” (*p* = .001), “trouble with social eating” (*p* = .001), “trouble with social contact” (*p* = .001), and “teeth” (*p* = .001). Patients who developed a recurrence did not show any significant higher (worse) scores in OHIP G14 or EORTC QoL-H&N35 scores compared with patients without recurrence. Twenty-nine out of 36 patients showed full mucosal healing (T2). For patients with no full mucosal healing, a downgrade to stage I was achieved.

**Conclusion:**

In terms of QoL patients with stage III MRONJ do benefit from surgical treatment. The incident of a recurrence seems to have no significant impact on patients QoL.

## Introduction

Since the first appearance in 2003 medication-related osteonecrosis of the jaw (MRONJ) remains challenging for clinicians and patients [1]. With the growing number of drugs causing MRONJ and the still not fully resolved pathology, it is part of ongoing controversies. Current treatments rely on position papers as there is no international standard guideline available until today [2–5]. Especially the treatment of stage III patients has shown to be a major challenge because despite the fact that patients benefit from surgical intervention, the risk of recurrence remains high and it might take more than one attempt to aim full mucosal healing [6–8]. Patients often suffer from pain, impairment of swallowing, or even a feeling of uncertainty regarding their teeth. This affects the quality of life (QoL) and increases with worsening stage [9]. Since a high level of QoL is a major goal of MRONJ treatment, it should be involved in treatment decision making. Unfortunately, there is very little known about the impact of surgical intervention in patients’ QoL especially when it comes to stage III MRONJ [10]. The aim of this study was to determine potential change in stage III MRONJ patients’ QoL after surgery. The null hypothesis was no significant change in QoL after surgical intervention. The specific aims of the study were (1) to measure patients’ QoL over time in order to identify which parts of QoL were affected by the disease and whether or not it changed after surgery, (2) to detect the impact of a recurrence on the change in QoL, and (3) to estimate the effect of covariates such as age, sex, medication, duration of medication, location of MRONJ, and risk factors.

## Materials and methods

### Study design and sample

To answer the research question, we designed and implemented a prospective monocentric cohort study (Department of Oral and Maxillofacial Surgery, University Hospital Regensburg Germany). The study was approved by the local ethical committee (Nr. 16-101-0257). Over a period of more than two and a half years (September 2016 to March 2019), patients with an established diagnosis of stage III MRONJ were included. MRONJ was diagnosed and classified according to the American Association of Oral and Maxillofacial Surgeons (AAOMS). The inclusion criteria were “exposed and necrotic bone or a fistula that probes to bone in patients with pain, infection, and one of the following: exposed and necrotic bone extending beyond the region of alveolar bone (i.e., inferior border and ramus in mandible, maxillary sinus, and zygoma in maxilla) resulting in pathologic fracture, extraoral fistula, oral antral or oral nasal communication, or osteolysis extending to inferior border of the mandible or sinus floor“ [2]. Patients were excluded from the study when they had a history of radiation therapy to the head and neck area, exposed bone, or fistula persisted less than 8 weeks or they showed obvious metastatic disease to the jaw.

### Variables

The primary predictor variable was time of evaluation. The QoL questionnaires were answered preoperative (T0), 6 weeks postoperative (T1), and 6 months postoperative (T2).

Primary outcome variables were QoL measures. The QoL was assessed by using two established measures: the European Organisation for Research and Treatment of Cancer QoL-H&N35 (EORTC QoL-H&N35) and the Oral Health Impact Factor-G14 (OHIP-G14) questionnaire. The EORTC QoL-H&N35 contains 35 questions assessing symptoms and side-effects of treatment, social function, body image, and sexuality. It contains seven multi-item scales as well as eleven single item measures. All multi item-scales contain a different set of items with no item occurring in more than one scale (Table 1). The given answers (1 “not at all” to 4 “very much” or yes/no) were converted to a range from 0 to 100 and evaluated statistically. High scores represent a higher level of symptomatology [11, 12]. Since a number of studies using EORTC QoL-H&N35 had problems with missing data on question (Q) 29 and 30 regarding to the item “sexuality,” we decided to remove them from the survey [13–15]. We replaced them with two questions on the impact on daily life with the same range of answers. Q29 “do thoughts on your primary disease affect your everyday life?” Q30 “do you feel impaired to do physical work? (for example, household chores).” They were combined to the multi-item scale “impact on daily life.” The OHIP-G14 contains 14 questions referring to oral health-related quality of life. The values of the answers range from 1 “never” to 4 “very often” and were summed up to an additive-OHIP-G14 score. Those scores were statistically compared [16, 17].

**Table 1:** EORTC QoL-H&N35 Symptom scales/items

Symptom scales/items	Number of items	Item range*	QoL-H&N35 Items
Pain	4	3	1–4
Swallow	4	3	5–8
Sense problems	2	3	13–14
Speech problems	3	3	16,23,24
Trouble with social eating	4	3	19–22
Trouble with social contact	5	3	18,24,28
Sexuality/Impact on daily life**	2	3	29,30
Teeth	1	3	9
Opening mouth	1	3	10
Dry mouth	1	3	11
Sticky saliva	1	3	12
Coughing	1	3	15
Felt ill	1	1	17
Nutritional supplements	1	1	31
Feeding tube	1	1	32
Weight loss	1	1	33
Weight gain	1	1	34

*“Item range” is the difference between the possible maximum and minimum value of individual items

**Questions on “sexuality” were changed to questions on “impact on daily life”

Other variables were anatomic location of exposed bone or fistula (upper or lower jaw), age (≥ 63 <years), sex, duration of medication (time from first intake to last intake or first hospitalisation regarding MRONJ in months), dental extraction prior MRONJ, and smoking (present-yes/no).

The secondary outcome variable was the appearance of a recurrence.

### Data collection methods

All patients were treated with surgical intervention performed under general anaesthesia using nasal intubation. After dissection of a mucoperiosteal flap necrotic bone was resected with a bone saw and piezo surgery. Sharp bone edges were smoothened till visible bleeding was reached. The tension-free and saliva-tight wound closure was accomplished with a multiple layer closure technique. All patients received therapeutic perioperative antibiotics starting 1 day before till 10 days after surgery. Amoxicillin/clavulanic acid was administered unless patients had a known allergy to penicillin. In that case clindamycin was administered. Food intake was ensured by a nasogastric feeding tube for 10 days. Antimicrobial mouth rinse with chlorhexidine (0.12%) was used 3 times a day. After patients were discharged from hospital; they had an examination at 14 days and 6 weeks after surgery. Afterwards, all patients were included in a routine 6-month follow-up program.

### Data analyses

Statistical analysis was performed using SPSS 26 (SPSS Inc., Chicago, IL, USA). Scores from the EORTC QoL-H&N35 survey were calculated based on the official scoring Manual [11]. Repeated measure analysis of variance (ANOVA) was performed to detect significant changes in the survey scores. A repeated measure analysis of covariance (ANCOVA) was executed to determine whether or not co-variables significantly affected the QoL. In cases of violation of sphericity, the Greenhouse-Geisser adjustment was used. Fisher exact test was used to evaluate whether patients who smoked before, during, and after the surgery are more likely to develop a recurrence. It was also used to evaluate the impact of the anatomic location on possible relapses. A *p* ≤ .05 was considered as statistically significant.

## Results

Forty-three patients with a stage III MRONJ and a mean age of 68 years (range 40–88) underwent surgical intervention. The mean duration of antiresorptive therapy was 63 months (range 3–423). Further patient characteristics are summarised in Table 2. The 6-week survey was accomplished by 43 patients. About 83.7% (36/43) of the patients completed the 6-month follow-up. Six patients passed away, and one patient felt unable to participate in the 6-month follow-up. The mean follow-up period was 21.86 weeks with a minimum of 6 weeks and a maximum of 6 months. About 34.9% (15/43) had dental extraction before MRONJ first occurred. After surgery 25.6% (11/43) of all patients developed recurrences within the first 6 months. Approximately 63.6% (7/11) of all relapses occurred within the first 6 weeks. About 42.9% (3/7) of those early relapses showed full mucosal healing up to the 6-month examination. Approximately 19.4% (7/36) of patients remained with a relapse even after the 6-month follow-up. Prior surgery 20.9% (9/43) of patients were smokers. Smokers showed a significant higher risk of developing a relapse that lasts longer than 6 months compared with non-smokers (*p* = .05). The location of the MRONJ did not show a significant impact on the risk of developing a relapse (T1 *p* = .624; T2 *p* = .652). Twenty-nine out of 36 patients showed full mucosal healing (T2). In case of non-full mucosal healing a stage improvement from stage III to I was achieved.

**Table 2:** Patients characteristics

Number of patients	43	
• Male	21	48.8%
• Female	22	51.2%
Primary malignant disease	36	83.7%
• Mamma carcinoma	11	30.6%
• Prostate carcinoma	14	38.9%
• Multiple myeloma	8	22%
• Lung carcinoma	2	5.6%
• Leiomyosarcoma	1	2.8%
Primary benign disease	7	16.3%
• Osteoporosis	7	100%
Oral bisphosphonate medication	6	14%
• Aledronic acid	6	100%
Intravenous bisphosphonate medication	33	76.7%
• Zoledronic acid	28	84.8%
• Pamidronic acid*	3	9.1%
• Ibandronic acid*	3	9.1%
Denosumab	13	30.2%
Bisphosphonate followed by Denosumab**	9	20.1%
Localisation	43	
• Upper jaw	9	20.9%
• Lower jaw	34	79.1%

*One patient received pamidronic acid followed by ibandronic acid

**If patients received bisphosphonate followed by denosumab they also appear as bisphosphonate or denosumab patients

One participant was excluded from all analysis regarding QoL (OHIP-G14 and EORTC QoL-H&N35) due to extreme values caused by an additional MRONJ stage II. It appeared in a different location than the previous stage III and therefore was not assessed as recurrence.

**Fig. 1:**
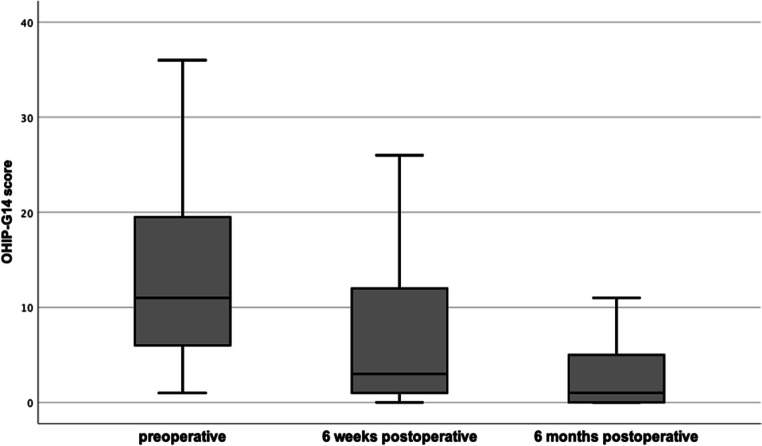
Comparison of OHIP-G14 scores (*n* = 35; preoperative: mean 13.40 SD 9.27; 6 weeks postoperative: mean 6.43 SD 7.19; 6 months postoperative: mean 2.80 SD 3.60). Means showed significant differences between each other (*p* < .001; *η*^2^ = .44)

**Fig. 2:**
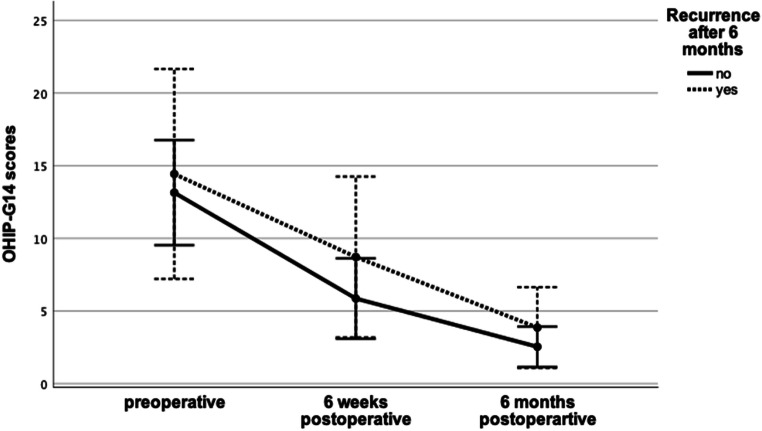
Comparison of OHIP-G14 scores between patients with and without recurrence after the 6-month follow-up. Groups show no significant differences (*p* = .85, *η*^2^ = .003)

OHIP-G14 scores decreased statistically significant (*n* = 35; after Bonferroni adjustment *p* = .001) from T0 (13.40 ± 9.27) to T1 (6.43 ± 7.19) by 52.02% and from T1 to T2 (2.80 ± 3.60) by 56.45% (Fig. 1). Co-variables had no significant impact on the improving OHIP-G14 scores over time (Table 3). Patients who developed a recurrence did not show significant (*p* = .181) differences in OHIP-G14 scores at any time, regardless if the recurrence occurred within the first 6 weeks (T1; *p* = .105) or between T1 and T2 (*p* = .820) (Fig. 2).

**Table 3:** Correlation between co-variables and the *p*value of OHIP-G14 scores after surgical treatment

Co-variables		*n*	Preoperative		6 weeks postoperative		6 months postoperative		*p* value	*η*^2^
			Mean	SD	Mean	SD	Mean	SD		
None		35	13.40	9.27	6.43	7.19	2.80	3.60	.001	.436
Age	≥ 68 years	16	14.81	10.13	7.38	7.78	2.81	3.25	.638	.011
	< 68 years	19	12.21	8.59	5.63	6.76	2.80	3.97		
Sex	Male	18	12.61	9.42	3.56	4.63	2.00	2.45	.255	.041
	Female	17	14.24	9.32	9.47	8.24	3.65	4.44		
Latest medication	Denosumab	10	15.70	11.17	6.90	8.33	3.10	4.23	.605	.013
	Bisphosphonates	25	12.48	8.49	6.24	6.86	2.68	3.41		
Duration of medication	≥ 63 m	10	16.20	10.33	10.10	9.33	3.90	4.46	.508	.018
	< 63 m	25	12.28	8.79	4.96	5.72	2.36	3.20		
Location of MRONJ	Upper jaw	8	12.00	8.88	9.13	8.61	4.00	4.44	.312	.034
	Lower jaw	27	13.81	9.51	5.63	6.69	2.44	3.33		
Smoking	Yes	9	11.44	6.46	8.00	7.86	2.89	3.62	.364	.029
	No	26	14.08	10.09	5.88	7.02	2.77	3.67

EORTC QoL-H&N35 scores were statistically compared for T0, T1, and T2. Results are shown in Table 4. The decrease of the score was significant after Bonferroni adjustment for following symptom scales/items: “swallowing” (*p* = .007), “opening mouth” (*p* = .045), “painkiller” (.005), “weight loss” (.004), “pain” (*p* = .001), “trouble with social eating” (*p* = .001), “trouble with social contact” (*p* = .001), and “teeth” (*p* = .001). None of the EORTC QoL-H&N35 symptom scales/items showed significant differences between patients with and without recurrence. None of the symptom scales/items showed significant interactions with any of the tested co-variables. Correlation between co-variables and the *p* value of EORTC QoL-H&N35 scores for those symptom scales/items that improved significantly after surgery are shown in Table 5.

**Table 4:** EORTC QLQ-H&N35 scores

Symptom scale/item	Preoperative		6 weeks postoperative		6 months postoperative		Δ%	*p* value	*η*^2^
	Mean	SD	Mean	SD	Mean	SD			
Pain	28.10	24.68	9.52	14.73	2.14	4.21	-92.38	.001	.433
Swallowing	10.71	17.10	4.52	7.38	2.38	5.18	-77.78	.007	.165
Senses problems	8.57	25.68	5.24	17.04	10.00	20.29	+16.69	.270	.038
Speech problems	5.71	13.84	6.67	18.90	0.95	4.15	-83.36	.133	.059
Trouble with social eating	24.52	26.50	13.36	20.07	4.76	8.16	-80.59	.001	.263
Trouble with social contact	9.52	15.28	4.95	12.66	0.38	2.25	-96.01	.001	.194
Impact on daily life	40.48	31.65	32.85	28.15	31.43	31.51	-22.36	.190	.049
Teeth	29.52	35.95	6.67	17.71	2.86	9.47	-90.31	.001	.317
Opening mouth	17.14	35.58	5.71	17.12	2.86	9.47	-83.31	.045	.102
Dry mouth	30.48	39.08	23.81	34.84	20.95	30.34	-31.27	.228	.043
Sticky saliva	18.10	29.53	12.38	24.37	11.43	24.18	-36.85	.209	.046
Coughing	14.29	31.61	8.57	21.91	7.62	16.34	-46.68	.327	.032
Felt ill	21.91	34.25	19.05	29.47	14.29	25.93	-35.05	.513	.019
Pain killers	65.71	48.16	34.29	48.16	42.86	50.20	-34.77	.005	.146
Nutrition supplements	11.43	32.28	11.43	32.28	8.57	28.40	-25.02	.898	.003
Feeding tube	0.00	0.00	0.00	0.00	0.00	0.00	0.00	-	-
Weight loss	37.14	49.02	11.43	32.28	8.57	28.40	-76.93	.004	.153
Weight gain	8.57	28.40	20.00	40.58	14.29	35.50	+66.75	.373	.029

**Table 5:** Correlation between co-variables and the *p*-value of EORTC QOL-H&N35 scores after surgical treatment

Symptom scale/item	None	Age	Sex	Latest medication	Duration of medication	Location of MRONJ	Smoking
		≥ 68 year/< 68 year	male/female	Denosumab/Bisphosphonates	≥ 63 m/< 63 m	Upper/lower jaw	Yes/No
Pain	.001	.712	.481	.561	.650	.649	.535
Swallowing	.007	.501	.139	.097	.935	.447	.584
Trouble with social eating	.001	.136	.800	.527	.326	.641	.403
Trouble with social contact	.001	.743	.782	.230	.837	.329	.321
Teeth	.001	.428	.536	.258	.241	.483	.119
Opening mouth	.045	.703	.364	.480	.751	.229	.566
Pain killers	.005	.467	.168	.270	.239	.841	.702
Weight loss	.004	.856	.380	.871	.175	.408	.638

## Discussion

The aim of this study was to show the impact of surgical treatment on patients suffering from MRONJ stage III in terms of quality of life. The hypothesis was that patients do profit from surgical intervention. Apart from the change in QoL the other special aims were to evaluate the influence of a recurrence on the process. Furthermore, the impact of co-variables was analysed. By assessing the QoL of 43 patients prior to 6 weeks (*n* = 43) and 6 months (*n* = 36) after surgery, we were able to show a significant improvement of the oral health impact factor in general (OHIP-G14) and in some parts of the overall quality of life (EORTC QoL-H&N35).

In cases where long-term wound closure was not obtained, the improvement of QoL did not significantly differ from those patients with full mucosal healing. This might be owed to the fact that even if patients developed a recurrence the MRONJ stage at least improved from stage III to stage I which includes the absence of symptoms and no evidence of infection [3]. That downgrading to stage I appeared to be important since the highest decrease in QoL occurs between stage I and stage II [9]. In conclusion, it can be assumed that although the primary goal is to achieve full mucosal healing, the experienced enhancement in terms of QoL is already accomplished by improving from MRONJ stage III to I. The improvement in QoL between a patient with stage 0 and a patient persisting with stage I remains low. None of the co-variables (age, sex, medication, duration of medication, localisation, smoking) showed significant impact on patients’ QoL at any time.

With 29 out of 36 patients showing full mucosal healing at the 6-month follow-up, our treatment results are comparable with current studies which mention a full mucosal healing rate of 85% in MRONJ stage III patients [18]. In all studies invasive surgery without microvascular flap reconstruction was performed [7, 19–23]. Tooth extraction prior to MRONJ diagnosis was reported for more than one third of the patients. Studies have shown that this is a common predisposing event. [24–26]. On the contrary Otto et al. has described that not the procedure of extraction leads to the development of MRONJ but rather a prevailing infectious condition in the bone that may increase the risk. By observing treatment protocols which include perioperative antibiotic prophylaxis, atraumatic surgery, smoothening of sharp bony edges, and saliva tight wound closure, tooth extractions can be performed safely [27]. Due to the fact that none of the tooth extractions were performed by the doctors of our department, we cannot evaluate whether or not those suggestions were obtained. In the first 6 weeks after surgery smoking had no significant impact on the risk of developing a recurrence. On the contrary after 6 months it was more likely for smokers to remain with exposed bone. This leads us to the assumption that not smoking itself triggers the occurrence of relapses in a significant way but rather suppresses the secondary wound healing process of a persistent relapse [28]. We presume that apart from new necrotic bone one major reason for a higher risk of recurrence is that a tension free wound closure was not obtained.

The QoL appeared to be mainly affected by two different aspects. One major factor was “pain.” Even though some patients did not feel any pain the majority suffered from constant pain which increased while eating. As a result, we observed high scores (low level of QoL) in “swallowing” which include problems with chewing and swallowing soft or solid food. In some cases, patients had to interrupt eating because of an aching jaw. This suggests an influence on the high level of “weight loss” combined with nearly no “weight gain” before surgery. After surgery “pain” scores decreased and so did “swallowing” problems. In consequence of less eating problems “weight loss” also showed lower scores with higher scores in “weight gain.” The enhancement was also apparent by the usage of pain killers. Although there was significant decrease in painkillers intake, the consumption remained high. At this point we did not have information about the dosage of painkillers which limits the validity. Furthermore, patients suffered from an underlaying disease which often caused remaining pain independent from MRONJ.

The mental health seemed to be the second major aspect influencing the QoL. Patients described a feeling of uncertainty regarding to their teeth. In addition to that some patients complained about foetor ex ore. This uncomfortable feeling in combination with insufficient dentition or bad/non fitting denture due to MRONJ led to “troubles with social eating” and even “troubles with social contact.” Surgery helped to overcome these difficulties in patients’ every day social life. In some cases, it appeared that the event of getting new prosthesis had an essential impact on how patients experienced their current situation. The question when to get new dental prosthesis was frequently asked. Depending on the location and the healing progress, we suggested to wait at least 6 weeks after surgery.

To our knowledge, this is the first study that examines the change in QoL after surgical treatment for a specific MRONJ stage (stage III). So far there are only two studies that determine the change in QoL. None of them differentiates between MRONJ stages [29, 30]. One weakness of our study is the low number of participants. This is owed to the small incidence of MRONJ [31]. Although we were able to determine a significant improve in terms of QoL in general, the small number of patients makes subgroup analysis unreliable. Another weakness is that we were not able to compare our findings with a control group since there is none available based on participants with similar underlying diseases. When considering the fact that QoL is primarily dependent on the current MRONJ stage, further analysis regarding QoL should be distinguished by MRONJ stage in order to make comparisons across studies more significant and to improve the practicability in everyday use.

## Conclusions

Our findings suggest that patients suffering from stage III MRONJ do benefit from surgical treatment. The quality of life improved significantly over time. After treatment patients who developed a recurrence (stage I) did not show different quality of life scores than patients with full mucosal healing. Nevertheless, further studies are necessary to evaluate the impact of different therapeutic approaches on patient’s quality of life especially in advanced MRONJ stages.

## Data Availability

Not applicable
