# Correction to: Patients’ quality of life improves after surgical intervention of stage III medication-related osteonecrosis of the jaw

**DOI:** 10.1007/s10006-021-01018-x

**Published:** 2021-11-29

**Authors:** Stefan Moll, Steffen Mueller, Johannes K. Meier, Torsten E. Reichert, Tobias Ettl, Christoph Klingelhöffer

**Affiliations:** Department of Cranio- and Maxillofacial Surgery, Hospital of the University of Regensburg, Franz-Josef-Strauß-Allee 11, 93053 Regensburg, Germany

**Correction to: Oral and Maxillofacial Surgery (2021) 25:359–366**

10.1007/s10006-020-00927-7

The article “Patients’ quality of life improves after surgical intervention of stage III medication-related osteonecrosis of the jaw”, written by Stefan Moll, Steffen Mueller, Johannes K. Meier, Torsten E. Reichert, Tobias Ettl and Christoph Klingelhöffer, was originally published Online First without Open Access. After publication in volume 25, issue 3, page 359–366 the author decided to opt for Open Choice and to make the article an Open Access publication. Therefore, the copyright of the article has been changed to © The Author(s) 2020 and the article is forthwith distributed under the terms of the Creative Commons Attribution 4.0 International License, which permits use, sharing, adaptation, distribution and reproduction in any medium or format, as long as you give appropriate credit to the original author(s) and the source, provide a link to the Creative Commons licence, and indicate if changes were made. The images or other third party material in this article are included in the article’s Creative Commons licence, unless indicated otherwise in a credit line to the material. If material is not included in the article’s Creative Commons licence and your intended use is not permitted by statutory regulation or exceeds the permitted use, you will need to obtain permission directly from the copyright holder. To view a copy of this licence, visit http://creativecommons.org/licenses/by/4.0.

The original article has been corrected.

